# Prospective and External Validation of Machine Learning Models for Short- and Long-Term Mortality in Acutely Admitted Patients Using Blood Tests

**DOI:** 10.3390/jcm13216437

**Published:** 2024-10-27

**Authors:** Baker Nawfal Jawad, Izzet Altintas, Jesper Eugen-Olsen, Siar Niazi, Abdullah Mansouri, Line Jee Hartmann Rasmussen, Martin Schultz, Kasper Iversen, Nikolaj Normann Holm, Thomas Kallemose, Ove Andersen, Jan O. Nehlin

**Affiliations:** 1 Department of Clinical Research, Copenhagen University Hospital Amager and Hvidovre, 2650 Hvidovre, Denmarkjan.nehlin@regionh.dk (J.O.N.); 2 Department of Clinical Medicine, University of Copenhagen, 2200 Copenhagen, Denmark; 3 Emergency Department, Copenhagen University Hospital Amager and Hvidovre, 2650 Hvidovre, Denmark; 4 Department of Cardiology, North Zealand Hospital, 3400 Hillerød, Denmark; 5 Emergency Medical Services, Capital Region, 2750 Ballerup, Denmark; 6 Department of Geriatrics, Copenhagen University Hospital Amager and Hvidovre, 2650 Hvidovre, Denmark; 7 Department of Emergency Medicine, Copenhagen University Hospital Herlev and Gentofte, 2730 Herlev, Denmark; 8 Department of Cardiology, Copenhagen University Hospital Herlev and Gentofte, 2730 Herlev, Denmark; 9 Department of Applied Mathematics and Computer Science, Technical University of Denmark, 2800 Kongens Lyngby, Denmark

**Keywords:** machine learning models, mortality prediction, emergency department, clinical biochemistry, biomarkers, short-term mortality, long-term mortality, explainable artificial intelligence (XAI)

## Abstract

**Background**: Predicting mortality in emergency departments (EDs) using machine learning models presents challenges, particularly in balancing simplicity with performance. This study aims to develop models that are both simple and effective for predicting short- and long-term mortality in ED patients. Our approach uses a minimal set of variables derived from one single blood sample obtained at admission. **Methods**: Data from three cohorts at two large Danish university hospitals were analyzed, including one retrospective and two prospective cohorts where prognostic models were applied to predict individual mortality risk, spanning the years 2013–2022. Routine biochemistry analyzed in blood samples collected at admission was the primary data source for the prediction models. The outcomes were mortality at 10, 30, 90, and 365 days after admission to the ED. The models were developed using Light Gradient Boosting Machines. The evaluation of mortality predictions involved metrics such as Area Under the Receiver Operating Characteristic Curve (AUC), sensitivity, specificity, negative predictive values, positive predictive values, and Matthews correlation coefficient (MCC). **Results**: A total of 43,648 unique patients with 65,484 admissions were analyzed. The models showed high accuracy, with very good to excellent AUC values between 0.87 and 0.93 across different time intervals. **Conclusions**: This study demonstrates that a single assessment of routine clinical biochemistry upon admission can serve as a powerful predictor for both short-term and long-term mortality in ED admissions.

## 1. Introduction

Effectively identifying patients at low and high risk of adverse outcomes is crucial for optimal resource allocation and treatment prioritization in healthcare systems. With the global population aging [1], the demand on emergency departments (EDs) is expected to rise significantly [2,3]. Consequently, this underlies the urgent need for innovative, personalized care strategies to ensure efficient resource use and patient care. However, in advancing these novel strategies, finding a balance between simplicity and performance becomes critical. High-performance solutions often come with complexity that may limit their practicality in dynamic settings such as EDs. Addressing this challenge is critical to developing effective yet manageable tools that can adapt to the fast-paced nature of emergency care.

In clinical practice, predicting all-cause mortality and assessing risk have consistently been crucial outcomes for clinicians [4,5,6,7,8,9]. Various scores and indices based on simple linear relationships have been proposed and used to predict mortality; however, their accuracy is only moderate [4,8,10]. In recent years, the application of ML to healthcare data has surpassed these traditional methods, offering enhanced accuracy in predicting outcomes for various patient groups, including those with conditions such as cardiac disease [11], COVID-19 [12], trauma [13], sepsis [14], and those in intensive care units (ICUs) [15]. While ML algorithms offer significant advantages, their integration into clinical practice presents challenges, primarily due to the incorporation of numerous clinical and nonclinical variables. This complexity poses a challenge for clinicians in terms of comprehension and practical application within the fast-paced environment of Eds [16]. Despite advancements in ML for mortality prediction, significant research gaps persist: most models are either designed as triage tools for short-term outcomes or as risk assessment tools for long-term mortality, typically focusing on specific patient cohorts. Additionally, there is a lack of models that address both short- and long-term outcomes simultaneously across a diverse patient population, and few of these models are interpretable. The effectiveness of ML models in clinical settings is closely linked to their transparency and interpretability, highlighting the need for predictive and comprehensible models for clinicians.

Recent developments in deep learning, particularly Transformer-based models, have shown great promise in medical data [17,18]. However, based on the better interpretability [19,20] and superior performance demonstrated in our previous findings [21], we opted to use the LightGBM algorithm. In this study, we aimed to develop and externally validate an easily adaptable prognostic machine learning tool, the short- and long-term mortality models (SLTM), which are designed to predict both short-term and long-term mortality among patients acutely admitted to the ED. These models utilize a single blood sample for routine clinical biochemistry analysis, including biomarkers of vital organs and the immune system. We also aimed to incorporate explainable ML techniques to more clearly explain how the models use input variables to make predictions, thereby assisting clinicians in understanding the ML model’s predicted outcomes.

## 2. Materials and Methods

### 2.1. Study Design and Settings

In this study, we evaluated data from three study cohorts. First, the retrospective 29K cohort study from the ED at the Copenhagen University Hospital Amager and Hvidovre (AHH), Denmark, was included. The 29K cohort included all patients admitted to the acute medical unit with an available blood sample. The 29K cohort consisted of 51,007 patient records from ED admissions between 18 November 2013 and 17 March 2017. The acute medical unit at the ED receives patients across all specialties, except for children (patients under 18 years), gastroenterological patients, and obstetric patients. Second, a prospective observational cohort, the TRIAGE Study, from the ED at North Zealand University Hospital (NZH), Hilleroed, Denmark, was included [22]. The TRIAGE cohort included all patients admitted to the ED with an available blood sample and consisted of 6383 patient records from ED admissions between 5 September 2013 and 6 December 2013 [22]. Children and obstetric patients were excluded. Third, a prospective observational cohort from the specialized COVID-19 Unit of the ED at AHH was included. This RESPOND-COVID cohort (Respiratory Emergency Surveillance and suPAR Outcome in COVID and Non-COVID Disease) included patients admitted with respiratory symptoms and suspected of having COVID-19. The RESPOND-COVID cohort consisted of 28,210 patient records from admissions between 10 March 2020 and 31 March 2022. The follow-up for vital status data was retrieved from the Central Public Server at Statistics Denmark and the Danish Civil Registration System, which record the status of all Danish residents. Identification was through the Central Personal Registry (CPR) number, unique to each citizen. During the study period, patients who left the country had their last admission data censored.

This study was reported in accordance with the transparent reporting of a multivariable prediction model for individual prognosis or diagnosis (TRIPOD) statement [23]. The research conducted in this study was in strict compliance with both regional and national guidelines and received the necessary approvals from the appropriate Danish authorities. The Danish Data Protection Agency (29K: HVH-2014-018, 02767; TRIAGE: J. 2007-58-0015) and the Danish Health and Medicines Authority (29K: 3-3013-1061/1; TRIAGE: 31-1522-102) approved the studies and analyses. Furthermore, the Legal Office at the Capital Region of Denmark, specifically the Team for patient medical records, issued a permit with reference numbers R-22041261 and R-20064514 for the usage and management of healthcare data within the region. The study adhered to all relevant ethical and legal standards required for research within Denmark.

### 2.2. Medical Records

Information regarding hospital admissions and discharges for the 29K cohort was extracted from the Danish National Patient Registry (NPR). In the RESPOND-COVID and TRIAGE cohorts, information related to patients’ health conditions, vital signs, triage, events in-hospital requiring continued care at the ED, and duration of hospital stay was retrieved from the patient’s health records in OPUS Arbejdsplads (version 2.5.0.0 Computer Sciences Corporation [CSC], Falls Church, VA, USA) and “Sundhedsplatformen” (Epic, version 2016, Verona, WI, USA). The TRIAGE study also involved the review of each patient record by two experts in internal or emergency medicine, who evaluated whether each admission was necessary or could have been avoided. An admission was deemed unnecessary only if both specialists agreed on this assessment. These reviewers were blinded to their own roles as either primary or secondary evaluators of each case and to the other reviewer’s decisions. An unnecessary admission was defined as one where the patient’s condition could have been adequately managed by a general practitioner or in an outpatient setting within 1–2 weeks. For all three cohorts, blood test results were obtained from the LABKA II national database [24]. Using each person’s unique personal identification number from the Danish Civil Registration System, we linked the data, encompassing biochemistry, diagnoses, records of hospital admissions, readmissions, and mortality.

### 2.3. Biomarkers

For all three cohorts, blood samples were collected upon admission to the ED, and routine whole blood counts along with clinical biochemistry were analyzed by AHH’s and NZH’s respective Departments of Clinical Biochemistry. The results were extracted from the LABKA database. The admission blood tests included C-reactive protein (CRP), soluble urokinase plasminogen activator receptor (suPAR), alanine aminotransferase (ALAT), albumin (ALB), International Normalized Ratio (INR), coagulation factors 2, 7, 10 (KF2710), total bilirubin (BILI), alkaline phosphatase (ALP), creatinine, lactate dehydrogenase (LDH), blood urea nitrogen (BUN), potassium (K), sodium (NA), estimated glomerular filtration rate (eGFR), hemoglobin (HB), and counts of leukocytes, lymphocytes, neutrophils, monocytes, thrombocytes, eosinophils, and basophils. All procedures were executed following the appropriate guidelines and standards.

### 2.4. Outcomes

In this study, the outcomes were 10-, 30-, 90-, and 365-day mortality after admission at the ED. We define short-term mortality as death within 30 days, intermediate-term mortality as death within 90 days, and long-term mortality as death within 365 days.

### 2.5. Data Preparation

A standard format was applied to all data. Patient admissions with more than 50% of the data (routine clinical biochemistry results) missing were dropped. The median percentage of missing blood biochemistry results for each study was: 29K: 2.6%; TRIAGE: 3.0%; and RESPOND-COVID: 2.4%, with an overall interquartile range (IQR) ranging from 1.6% to 7.7%.

The 29K cohort was split into training and test sets. To avoid information leakage, as some patients were readmitted multiple times, we ensured that the same patient ID did not appear in both training and test sets. The TRIAGE and RESPOND-COVID datasets were exclusively used as test data.

To handle missing values, we employed iterative imputations from the Python scikit-learn package, version 1.5.2 [25]. This method was chosen due to its ability to impute missing values by modeling each feature as a function of the others iteratively, which can improve the accuracy of the imputation compared to simpler methods such as mean or median imputation. The imputation model used all available features as predictors to minimize data loss. Training and test sets were imputed separately to avoid data leakage. To validate the quality of our imputation process, we conducted a sensitivity analysis comparing models trained on imputed versus non-imputed data. The performance metrics showed minimal differences, suggesting that imputation did not significantly impact the result.

To handle class imbalance in our target outcome, we addressed imbalanced data by exploring several resampling methods, including random undersampling, random oversampling, and SMOTE. We ultimately chose random oversampling technique from the Imbalanced-Learn Package (Python), version 0.12.4 [26] during training. To reduce the impact of magnitude on the variance, we normalized the values of all variables in the data by the z score. This process was applied to the training data. For the test data, normalization was based on the mean and standard deviation estimated from the training data. To achieve a normal distribution approximation for our variables, we employed the Yeo-Johnson transformation [27] on the training data. For the test data, the transformation was based on the estimates derived from the training set. These preprocessing steps significantly enhanced the model’s performance on the validation set.

### 2.6. Model Construction

All the models were crafted using Python (version 3.8.0). We employed the classification module from PyCaret (version 2.2.6) [28] to develop models using four distinct algorithms for predicting 10-, 30-, 90-, and 365-day mortality. PyCaret is a low-code machine learning library that streamlines the entire machine learning process.

For hyperparameter optimization, we used the tune_model() function within PyCaret. The tuning process was configured to perform 100 iterations, optimizing the models based on the AUC metric. We selected Bayesian optimization, a search approach that chooses hyperparameters by learning from previous evaluations to improve future selections, as the search algorithm, leveraging the scikit-optimize library. To enhance computational efficiency, early stopping was implemented using the Asynchronous Successive Halving Algorithm. This approach allows for the early termination of underperforming models during the hyperparameter search process. The parameter choose_better = True was specified to ensure that the best-performing model, according to the AUC metric, was selected.

### 2.7. Algorithm Selection and Performance Measures

Fifteen machine learning algorithms were trained and evaluated using PyCaret through 10-fold cross-validation (Random Forest (RF), SVM-Radial Kernel (RBFSVM), Extra Trees Classifier (ET), Extreme Gradient Boosting (XGBOOST), Decision Tree Classifier (DT), neural network (MLP), Light Gradient Boosting Machine (LIGHTBM), K Neighbors Classifier (KNN), Gradient Boosting Classifier (GBC), CatBoost Classifier (CATBOOST), Ada Boost Classifier (ADA), Logistic Regression (LR), Linear Discriminant Analysis (LDA), Quadratic Discriminant Analysis (QDA), and Naive Bayes (NB)) [21].

These models were developed using fifteen biomarkers (ALAT, ALB, ALP, BUN, creatinine, CRP, eosinophils, KF2710, leukocytes, LDH, lymphocytes, neutrophils, platelets, sodium, and suPAR) from a single routine blood test, along with age and sex as additional variables. The feature selection was informed by previous work (Baker et al., 2014). However, in the TRIAGE cohort, mean corpuscular volume (MCV) and mean corpuscular hemoglobin concentration (MCHC) were not available. Therefore, based on the findings from Baker et al., 2024 [21], new models were trained using the top fifteen biomarkers, excluding MCV and MCHC.

The best machine learning algorithm (Light Gradient Boosting Machine [LIGHTGBM]) was fine-tuned and evaluated through a 10-fold cross-validation on the 29K cohort, then tested on an unseen test sample of the same cohort and on TRIAGE and RESPOND-COVID data. Model selection was based on the area under the receiver operating characteristic curve (AUC). Additionally, the sensitivity, specificity, positive predictive value (PPV), negative predictive value (NPV), and Matthew’s correlation coefficient (MCC) for the complete data were estimated for the validation and test data and evaluated between them. To evaluate the reliability of our predictive model’s performance, we used a bootstrap resampling technique to calculate confidence intervals (CIs) for key metrics, including AUC, sensitivity, specificity, PPV, NPV, and MCC. We performed 1000 bootstraping iterations.

### 2.8. Calibration

To improve our predictive model’s ability to estimate probabilities that accurately reflect actual outcomes, we implemented probability calibration using isotonic regression from the scikit-learn package (version 1.4) in Python [25]. Calibration was performed on unseen test data to prevent overfitting and ensure unbiased adjustments to the predicted probabilities.

To evaluate the model’s precision, we employed the Brier score as an accuracy metric, comparing its values before and after the calibration process. We observed that the Brier score improved after calibration, indicating that the calibrated model’s probability estimates were more accurate.

As expected, the AUC remained stable after calibration for all outcomes, indicating that the model’s ability to discriminate between classes did not change. However, when using the default threshold of 0.50, we observed notable shifts in sensitivity and specificity. Calibration impacted the probability estimates, leading to the default threshold no longer providing an optimal balance between sensitivity and specificity. To optimize the decision threshold for the best balance between sensitivity and specificity, we employed the Youden Index on the calibrated probabilities for all outcomes. This method empirically determines the optimal cutoff point post-calibration to achieve an optimal tradeoff between sensitivity and specificity.

### 2.9. Explaining Model Predictions

We used TreeExplainer, version 0.45.1 [29], a method based on the approximation of the set of trees to calculate the SHapley Additive exPlanations (SHAP) [30] values, to interpret our machine learning models’ predictions, quantifying the impact of each variable on individual predictions. SHAP values were calculated using the SHAP library package in Python, which helped assess variable contributions in terms of magnitude and direction. To visualize these contributions, we employed waterfall plots. These plots illustrated the influence of individual variables on individual model predictions.

### 2.10. Statistical Analysis

Statistical analyses were performed using R (version 4.1.0) and Python (version 3.8.0). Categorical variables are presented as frequencies (N) and percentages (%), whereas continuous variables are represented by their median values and IQR. To evaluate the differences between datasets, we employed the Student’s *t* test, setting our significance level at 5% for continuous variables and the chi-square test for categorical variables.

## 3. Results

### 3.1. Description of the Cohorts Used in the Study

In this study, we included a total of 65,484 admissions from the EDs of two different Danish hospitals, the Copenhagen University Hospital Amager and Hvidovre (AHH) and the North Zealand University Hospital (NZH). From AHH we included ED data from both retro and prospective cohorts: 29K (2013–2017) and RESPOND-COVID (2020–2022), respectively, in our analysis. From NZH, we included ED data from a prospective cohort, TRIAGE (2013). The hospital and patient characteristics are summarized in Table 1.

**Figure 1:**
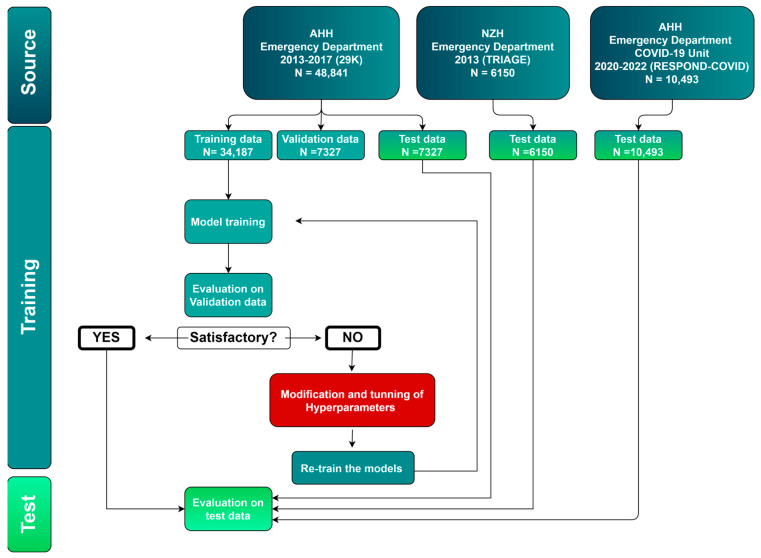
Flowchart of data training, validation, and testing using two ML algorithms. Patient medical records originated from three cohorts at two Danish hospitals: (1) 29K: Emergency Department at the Copenhagen University Hospital Amager and Hvidovre (AHH), 2013–2017, (2) TRIAGE: Emergency Department at North Zealand University Hospital (NZH), 2013, and (3) RESPOND-COVID: Emergency Department at AHH, 2020–2022. N = number of admissions.

For the 29K dataset, there were 51,007 admissions at the ED of AHH involving 28,683 unique patients during the study period. After excluding 2166 patient records for missing data, the study cohort consisted of 48,841 admissions from 28,671 unique patients (Figure 1). The cohort was herof named 29K. Of these, 34,187 (70%) were allocated as training data, 7327 (15%) as validation data, and 7327 (15%) as internal test data. The median age of the 29K patients at admission was 65.6 years (IQR: 48.2–78.5), with 52.3% being female.

The TRIAGE cohort from the NZH included 6383 admissions in the ED, involving 6356 unique patients. After excluding 233 patient records with missing data, the study cohort comprised 6150 admissions involving 6071 unique patients (Figure 1). All the TRIAGE data were used for external data validation. The median age of the TRIAGE patients at admission was 63.0 years (IQR: 46.0–76.0), with 50.6% being female.

The RESPOND-COVID cohort from AHH consisted of 28,210 patient records from 8853 unique patients; however, only 10,493 admissions from 8451 unique patients with a suPAR measurement were included. The median age of the RESPOND-COVID patients at admission was 66.0 years (IQR 49.1–78.2), with 51.2% being female.

Patients from the two AHH cohorts (29K and RESPOND-COVID) were slightly older (*p* < 0.0001), had a higher proportion of females (*p* < 0.01), and exhibited higher mortality rates (*p* < 0.0001), 4.0% and 4.4%, respectively, compared to patients from the NZH cohort, who had a mortality rate of 2.9% (Table 1). In general, the distributions of all the variables were significantly different among the three datasets. Patients excluded based on missing data from the 29K and TRIAGE datasets showed no significant differences compared to those included. However, in the RESPOND-COVID cohort, the excluded patients were predominantly older, mostly female, and had a lower mortality rate at 10 to 365 days.

### 3.2. Model Performance

**Figure 2:**
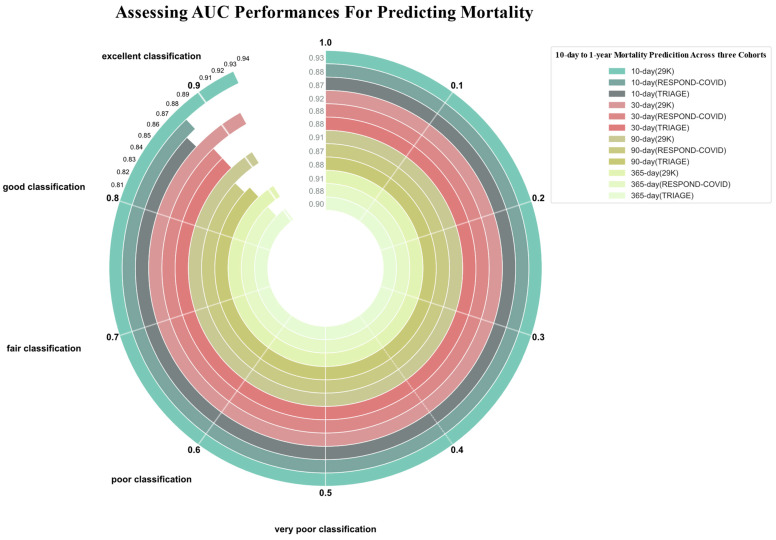
**Area under the receiver operating characteristic curve (AUC).** Performance for predicting 10-day (green shaded circles), 30-day (peach-shaded circles), 90-day (dark yellow shaded circles), and 365-day (light yellow shaded circles) mortality in each of the three cohorts, 29K, TRIAGE, and RESPOND-COVID, was examined.

Figure 2 illustrates the predictive performance of the LightGBM models, assessed by the AUC, for mortality predictions at 10, 30, 90, and 365 days. Additional performance metrics are detailed in Table 2. ROC curves for each time interval can be found in the Supplementary (Figures S1–S4). For the 29K test dataset, the LightGBM model demonstrated high predictive accuracy, exhibiting an AUC of 0.93 (95% CI: 0.92–0.94) for 10-day mortality predictions and an MCC of 0.30 (95% CI: 0.28–0.32). The model maintained high performance for 30-day mortality predictions with an AUC of 0.92 (95% CI: 0.90–0.92) and an MCC of 0.40 (95% CI: 0.38–0.42). For 90-day mortality, the AUC was 0.91 (95% CI: 0.90–0.92) alongside an MCC of 0.51 (95% CI: 0.49–0.53), and for 365-day mortality, the AUC was 0.91 (95% CI: 0.91–0.91), with an MCC of 0.53 (95% CI: 0.51–0.55).

In the RESPOND-COVID dataset, the AUCs were 0.88 (95% CI: 0.86–0.89) for the 10-day mortality prediction, 0.88 (95% CI: 0.87–0.89) for the 30-day mortality prediction, 0.87 (95% CI: 0.86–0.88) for the 90-day mortality prediction, and 0.88 (95% CI: 0.86–0.90) for the 365-day mortality prediction. The MCC values corresponded to 0.22 (95% CI: 0.20–0.24), 0.32 (95% CI: 0.30–0.33), 0.38 (95% CI: 0.36–0.40), and 0.43 (95% CI: 0.41–0.45), respectively. Lastly, for the TRIAGE dataset, the AUCs were 0.87 (95% CI: 0.85–0.89) for 10-day mortality, 0.88 (95% CI: 0.86–0.90) for 30-day, 0.88 (95% CI: 0.86–0.90) for 90-day, and 0.90 (95% CI: 0.89–0.91) for 365-day mortality. The MCCs were 0.25 (95% CI: 0.23–0.27), 0.34 (95% CI: 0.32–0.36), 0.40 (95% CI: 0.38–0.42), and 0.43 (95% CI: 0.41–0.45), respectively.

The calibrated LightGBM models, showed varying levels of sensitivity and specificity across the datasets for mortality prediction intervals (Table 2). In the 29K dataset, the sensitivity for predicting mortality ranged from 84–90%, while the specificity was between 79–83%. Within the RESPOND-COVID dataset, the model sensitivity was between 84–88%, with a specificity ranging from 70–78%. For the TRIAGE dataset, the sensitivity varied from 72–87%, and the specificity showed a narrow range of 75–84%.

### 3.3. Machine Learning Prediction Application

**Figure 3:**
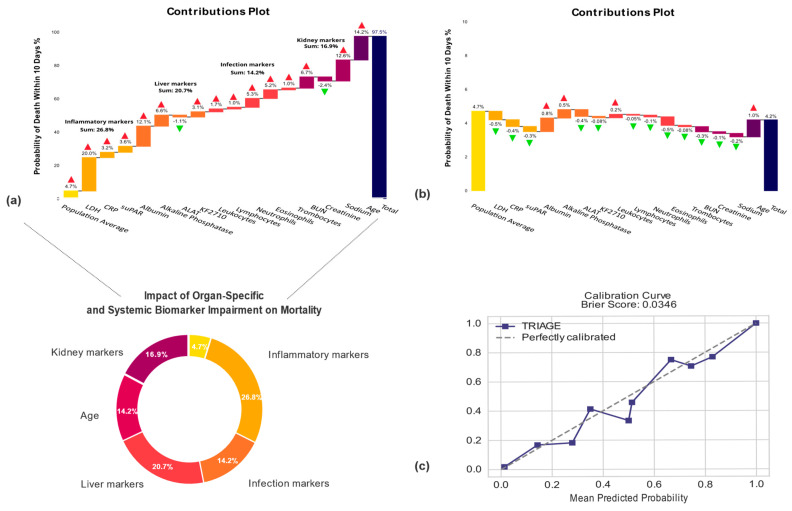
**The Light Gradient Boosting Machine (LIGHTBM) model’s individualized predictions of 10-day mortality risk for two specific patient cases in the prospective TRIAGE cohort.** In this analysis, we utilized explainable artificial intelligence (XAI) techniques, particularly those using estimated SHAP values, to analyze the LGBM model’s predictions of 10-day mortality risk for two specific patient cases within the TRIAGE cohort. These plots were based on the calibrated model (**a**). Case 1: a notable case of an elderly male patient who was admitted to the Emergency Department at North Zealand Hospital. *X*-axis: Displays the percentage contribution of biomarkers taken at the ED to the prediction of mortality risk. Below, these biomarkers are categorized by function: inflammatory, infection-related, liver, and kidney markers. (**b**) Case 2: This case involves an elderly female patient who presented at an Emergency Department in TRIAGE with a suspected infection. The model estimated a relatively low 10-day mortality risk of 4.2% for her. In case 1 and 2 variable sex is not shown (value 0.002) (**c**). Calibration plots on the TRIAGE cohort. *X*-axis: Mean Predicted Probability; *Y*-axis: Observed Frequency. The diagonal dash line stretching from the bottom left to the top right represents perfect agreement between the predicted probabilities and actual outcomes. The blue line is the model´s predicted probabilities and actual outcomes.

From our prospective cohort dataset TRIAGE at NZH, upon which we tested the prediction model, we analyzed a case of an elderly male patient with a history of cardiovascular and neurological conditions (Figure 3a). This patient was brought to the ED with symptoms suggesting an infection in the respiratory system. Upon arrival, the patient was categorized as having moderate urgency based on the triage (Early Warning Score). Laboratory tests revealed elevated levels of inflammatory markers, indicating a bacterial infection. The patient was treated with standard outpatient antibiotics and discharged the same day. Unfortunately, the patient’s condition deteriorated, leading to the patient’s passing a few days later. The short-term mortality model, predicting 10-day mortality, estimated a high mortality risk of 97.5% for this patient.

The contribution plot created using explainable artificial intelligence (XAI), depicted in Figure 3a, provides insights into how the patients impaired organ-specific biomarkers contributed to the model’s overall output of mortality risk. In this case, the most important contributors to mortality risk prediction were identified as markers of the inflammatory response to the infection (27%), markers of the immune system (14%), markers of liver function (21%), and kidney function (17%). These insights shed light on the specific factors driving the elevated risk for this patient. With this knowledge, this patient should have been hospitalized. Subsequent independent reviews by two specialists suggested that a hospital admission might have been necessary for this patient.

Moving to a different scenario, in another case, we examined an elderly female patient with a history of cardiovascular conditions and metabolic disease, presenting also with symptoms indicative of an infection in the respiratory system (Figure 3b). The patient was categorized as urgent upon triage. Laboratory tests indicated signs of infection, with elevated inflammatory markers and abnormal blood cell counts. Other notable laboratory results included imbalances in electrolytes and liver enzymes. Despite these findings, the LGBM model, analyzed through XAI, predicted a lower 10-day mortality risk of only 4.3%. The patient was hospitalized and treated with IV antibiotics for three days. However, subsequent reviews by specialists suggested that hospital admission might have been unnecessary.

## 4. Discussion

In this study, we utilized routine clinical biochemistry data from a single time point upon admission, representing vital organ and immune system function, to predict mortality risk in acutely admitted patients. By incorporating explainable ML methods, we ensured that the model’s outputs could be interpreted, thereby aiding clinicians in understanding the predicted ML outcomes. Our results for both short- and long-term mortality models demonstrated very good to excellent performance metrics, achieving high AUC values ranging from 0.87 to 0.93. Although a small decline in AUC values in the TRIAGE and RESPOND-COVID datasets was observed compared to the 29K dataset, this was anticipated due to significant differences in patient characteristics and mortality rates across cohorts. Performance metrics, especially AUC and MCC, showed overlapping confidence intervals for the RESPOND-COVID and TRIAGE datasets. This overlap indicates that the models performed similarly across these datasets. Nonetheless, we observed variability in the models’ sensitivity and specificity across the different cohorts.

Overall, the models demonstrated low PPVs ranging from 9% to 47%, indicating a large proportion of false positives, while showing very high NPVs ranging from 96% to 100%. A trend toward increases in PPV and MCC values was observed from short-term to long-term mortality prediction, indicating a higher probability to predict the outcome over the length of time. The low PPV in short-term mortality prediction could be attributed to the low mortality prevalence in the studied patient populations. Additionally, it is possible that the model identifies patients (false positive) as being at high risk of mortality, but upon readmission and/or subsequent treatment after their initial discharge, these patients survive. As regards the high NPV, the results should be interpreted considering the dataset’s overall low mortality rate, or conversely, its high survival rate.

In clinical practice, screening tools that offer high sensitivity and high NPV are preferred and well justified [31,32], as these tools align with clinicians’ needs for safely excluding individuals at low risk of adverse outcomes in the future. This approach is preferred due to the low pretest probability, and the goal of the diagnostic test will be “ruling out” the condition, emphasizing high sensitivity where a negative result effectively excludes the condition. This is in contrast with diagnostic tools, where a high pretest probability of a condition leads to the goal of “ruling-in” the condition, emphasizing high specificity and PPV, as a positive result effectively confirms the condition. Our ML models embody this clinical principle, providing reliable decision support that matches the preferences of healthcare practitioners. This alignment with clinical practices not only supports the utility of the models but also sets a foundation for their potential development and application in healthcare settings.

Comparing our models with existing ML models, in terms of short-term mortality prediction, our models achieved an AUC of 0.87 to 0.93 for 10-day mortality predictions across the studied cohorts. This performance, when compared to other promising models, seems to be either on par or clearly superior, as explained below. Nevertheless, it is crucial to acknowledge that comparing results from data across diverse populations can be complex, given the multifaceted nature of socioeconomic, health factors, and other variables. Furthermore, mortality rates can be different in each population. Despite these complexities, when reviewing the literature, we find notable results. For instance, Trentino et al. conducted a study analyzing data from three adult tertiary care hospitals in Australia [33]. This study achieved a remarkable AUC of 0.93 for predicting in-hospital mortality among all admitted patients, regardless of whether their cases were medical or surgical. The predictive model used in this study incorporated various variables, including demographics, diagnosis codes, administrative information, and the Charlson comorbidity index. Similarly, an ED triage tool, the Score for Emergency Risk Prediction (SERP), to predict mortality within 2 to 30 days for ED patients was initially applied in a cohort from a Singaporean ED and subsequently underwent external validation in a South Korean ED [34,35]. These studies demonstrated AUCs of 0.81–0.82 for in-hospital mortality and 0.80–0.82 for 30-day mortality prediction. The SERP scores incorporate variables, including age, vital signs, and comorbidities. Additionally, in a study conducted on hospitalized patients in the U.S. by Brajer et al., reported an AUCs between 0.86 and 0.89 based on 57 electronic health record data variables [36]. In contrast, our models performed competitively, achieving comparable or superior results for short-term mortality prediction using just 15 biomarkers measured from a single routine blood sample collected upon arrival in the ED. For 30-day mortality, our models consistently maintained high AUCs (0.88–0.92) in both the internal and external evaluations. Likewise, the long-term mortality models showed near-excellent performance, with AUCs ranging from 0.87 to 0.91 for 90-day mortality prediction and 0.88 to 0.91 for 365-day mortality prediction. The performance of this model is either superior or comparable to similar studies in the field.

The random forest models developed by Sahni et al. achieved an AUC of 0.86 for 1-year mortality prediction [37]. Their model incorporated various variables, including demographic, physiological, biochemical factors, and comorbidities. Similarly, Woodman et al. developed a ML model trained on a patient cohort aged > 65 years. Their model achieved an AUC of 0.76 and incorporated variables including demographic, BMI, anticholinergic risk score, biochemical markers, and a comprehensive geriatric assessment.

In this study, we have adopted a streamlined biomarker approach that aligns with the latest recommendations for AI deployment in healthcare settings, prioritizing consistency and reduced error susceptibility [38]. This approach, which is centered on a single blood sample routinely analyzed for a select set of standard vital organ and immune system biomarkers, presents significant advantages. Unlike existing tools that primarily focus on triage, our models extend their utility to encompass resource allocation, treatment planning, discharge, and potentially preventing overtreatment and ensuring that care aligns with the patient’s preferences and recovery potential. Specifically, it provides a stable and chronic disease-oriented perspective, which is crucial for uncovering underlying pathologies that might not be apparent with other data types.

In stark contrast to other models that depend on various inputs—such as continuous vital sign monitoring, administrative variables, medical history, comorbidities, and medication profiles—our model’s simplicity integrates more fluidly into clinical workflows and mitigates the ‘black box’ nature that often accompanies complex AI systems, where the intricacy of ML models and the use of non-clinical features can make it challenging to understand the rationale behind the model output. Our methodology, with its deliberately limited parameters, enhances the models’ output transparency and interpretability, thereby building confidence and trust among clinicians in AI-assisted decision-making.

### 4.1. Limitations

The exclusion of specific patient groups from the cohorts, including children and obstetric patients, limits the trained model applicability of our models to these populations. Furthermore, the retrospective design of our cohort introduces inherent limitations, such as the potential for selection and information biases. These biases can impact the validity of our findings and their applicability to broader, more diverse populations. There are also several limitations to the SHAP values. SHAP values are used for interpreting predictions of ML models, specifically by quantifying the contribution of each feature to a particular prediction. However, they do not provide causal insights. This means that while SHAP values can tell us which variables were important in the model’s decision-making process, they do not imply a cause-and-effect relationship between these variables and the prediction. Lastly, the models were primarily validated within the same geographical region and governing clinical jurisdiction. While they were evaluated across different cohorts, this regional focus might constrain the generalizability of our findings.

### 4.2. Future Research

The present models are only meant as a proof-of-concept study. Refining and validating these models with diverse datasets remains a priority. We also plan to perform further generalizability testing on external datasets from other countries and outpatient populations in future research to ensure applicability across different geographical regions and demographics. Future research should similarly focus on enhancing the PPV and incorporating more comprehensive patient data before implementation in clinical practice.

## 5. Conclusions

In this study, we have successfully developed and externally validated machine learning models that predict both short-term and long-term mortality in acutely admitted patients based on a single set of routine blood tests. With AUC scores ranging from 0.87 to 0.93, we have demonstrated that a simplified approach can achieve sufficient high predictive accuracy, with the potential to warrant investigation into its applicability as an additional tool in clinical decision-making.

## Figures and Tables

**Table 1:** Patient baseline characteristics and mortality rates.

Variable	Retrospective	Prospective	Prospective
Cohort	AHH (29K)	NZH (Triage)	AHH (RESPOND-COVID)
Year	2013–2017	2013	2020–2022
N of unique patients	28,671	6124	8451
N of admissions	48,841	6150	10,493
Demographics			
Age	65.6 (48.2–78.5)	63.0 (46.0–76.0)	66.0 (49.1–78.2)
Sex (female) ^6^	52.3%	50.5%	51.2%
Laboratory Biomarkers			
ALAT ^1^	21.0 (15.0–33.0)	20.8 (14.8–31.5)	23.0 (16.0–35.0)
Albumin ^2^	34.0 (30.0–37.0)	37.2 (33.5–39.8)	34.0 (30.0–37.0)
Alkaline Phosphatase ^1^	75.6 (63.0–94.0)	84.2 (69.3–105.9)	79.0 (63.0–103.0)
Bilirubin ^3^	7.0 (5.0–10.1)	7.9 (5.7–11.2)	8.0 (5.0–11.0)
BUN ^4^	5.1 (3.8–7.2)	5.2 (4.0–7.1)	5.3 (3.9–7.7)
Creatinine ^3^	77.0 (62.0–97.0)	71.0 (59.0–88.0)	77.0 (62.0–98.0)
CRP ^5^	7.0 (2.0–39.0)	5.2 (2.9–23.2)	12.0 (2.6–54.0)
HB ^4^	8.1 (7.2–8.9)	8.4 (7.6–9.0)	8.2 (7.3–9.0)
INR	1.0 (1.0–1.1)	1.0 (0.9–1.1)	1.0 (1.0–1.1)
Potassium ^4^	3.9 (3.6–4.2)	4.0 (3.8–4.3)	3.9 (3.6–4.2)
KF2710	0.9 (0.8–1.0)	0.9 (0.8–1.1)	0.8 (0.7–0.9)
LDH ^1^	186.0 (169.0–214.0)	182.6 (157.6–217.3)	214.0 (184.0–260.0)
Leukocytes ^6^	8.7 (6.9–11.3)	8.2 (6.5–10.6)	8.7 (6.6–11.8)
Lymphocytes ^6^	1.7 (1.1–2.3)	1.6 (1.2–2.0)	1.4 (0.9–2.1)
Monocytes ^6^	0.7 (0.5–0.9)	0.6 (0.5–0.8)	0.7 (0.5–0.9)
Neutrophils ^6^	5.8 (4.1–8.3)	5.7 (4.0–7.9)	6.0 (4.1–8.9)
suPAR (ng/mL)	3.3 (2.3–5.0)	4.5 (3.5–6.4)	4.1 (2.9–5.9)
Thrombocytes ^6^	247.0 (201.0–302.0)	238.0 (196.0–288.0)	248.0 (196.0–310.0)
Eosinophils ^6^	0.1 (0.0–0.2)	0.2 (0.1–0.4)	0.1 (0.0–0.2)
eGFR (mL/min)	80.0 (60.0–90.0)	86.3 (67.6–90.0)	77.0 (58.0–84.5)
Sodium ^4^	139.0 (136.0–141.0)	139.0 (136.9–140.6)	138.0 (135.0–140.0)
Mortality Rates			
Mortality rate 10 days ^6^	4.4% (1252)	2.9% (177)	4.0% (341)
Mortality rate 30 days ^6^	8.2% (2338)	4.6% (284)	8.4% (712)
Mortality rate 90 days ^6^	11.8% (3394)	7.8% (475)	12.4% (1052)
Mortality rate 1 year ^6^	16.3% (4677)	11.9% (729)	18.5% (1560)

The results are expressed as median (IQR, interquartile range) for continuous variables. For categorical variables, the results are expressed as the number of participants (percentage). ALAT: alanine-aminotransferase; BUN: blood urea nitrogen; CRP: C-reactive protein; eGFR: estimated glomerular filtration rate; HB: hemoglobin; INR: prothrombin time and international normalized ratio; KF2710: coagulation factors 2, 7, 10; LDH: lactate dehydrogenase; suPAR: soluble urokinase plasminogen activator receptor. 29K: Emergency Department at the Copenhagen University Hospital Amager and Hvidovre (AHH), 2013–2017. TRIAGE: Emergency Department at North Zealand University Hospital (NZH), 2013. RESPOND-COVID: Emergency Department at AHH, 2020–2022. ^1^: (U/L); ^2^: (g/L); ^3^: (µmol/L); ^4^: (mmol/L); ^5^: (mg/L); ^6^: number in (%).

**Table 2:** Results from test data for predicting short- and long-term mortality.

Test Data	N	AUC	Sensitivity	Specificity	PPV	NPV	MCC
10-day Mortality							
29K	7.327 (272)	0.93 (0.92–0.94)	0.90 (0.86–0.93)	0.82 (0.81–0.83)	0.12 (0.11–0.14)	1.0 (1.0–1.0)	0.30 (0.28–0.32)
RESPOND-COVID	10.493 (341)	0.88 (0.86–0.89)	0.88 (0.84–0.91)	0.70 (0.69–0.71)	0.09 (0.08–0.10)	0.99 (0.99–1.0)	0.22 (0.22–0.24)
TRIAGE	6.150 (177)	0.87 (0.85–0.89)	0.72 (0.65–0.79)	0.84 (0.84–0.85)	0.12 (0.10–0.14)	0.99 (0.99–0.99)	0.25 (0.22–0.29)
30-day mortality							
29K	7.327 (537)	0.92 (0.90–0.92)	0.89 (0.86–0.91)	0.83 (0.82–0.83)	0.23 (0.21–0.24)	0.99 (0.99–0.99)	0.40 (0.38–0.42)
RESPOND-COVID	10.493 (712)	0.88 (0.87–0.89)	0.89 (0.86–0.91)	0.68 (0.68–0.69)	0.18 (0.17–0.19)	0.99 (0.98–0.98)	0.32 (0.30–0.33)
TRIAGE	6.150 (284)	0.88 (0.86–0.90)	0.76 (0.71–0.81)	0.84 (0.83–0.85)	0.18 (0.16–0.21)	0.99 (0.98–0.99)	0.34 (0.30–0.37)
90-day Mortality							
29K	7.327 (982)	0.91 (0.90–0.92)	0.84 (0.82–0.86)	0.85 (0.84–0.86)	0.38 (0.36–0.40)	0.98 (0.98–0.98)	0.51 (0.49–0.53)
RESPOND-COVID	10.493 (1052)	0.87 (0.86–0.88)	0.84 (0.82–0.86)	0.73 (0.72–0.74)	0.28 (0.26–0.29)	0.97 (0.97–0.97)	0.38 (0.36–0.40)
TRIAGE	6.150 (475)	0.88 (0.86–0.90)	0.77 (0.73–0.81)	0.84 (0.83–0.85)	0.30 (0.27–0.32)	0.98 (0.97–0.98)	0.40 (0.37–0.43)
365-day mortality							
29K	7.327 (1812)	0.91 (0.91–0.91)	0.87 (0.86–0.88)	0.79 (0.79–0.79)	0.47 (0.46–0.48)	0.97 (0.96–0.97)	0.53 (0.51–0.55)
RESPOND-COVID	10.493 (1569)	0.88 (0.86–0.90)	0.85 (0.83–0.87)	0.78 (0.77–0.79)	0.45 (0.44–0.47)	0.96 (0.96–0.97)	0.43 (0.42–0.45)
TRIAGE	6.150 (729)	0.90 (0.89–0.91)	0.87 (0.84–0.89)	0.75 (0.74–0.76)	0.32 (0.30–0.34)	0.98 (0.97–0.98)	0.43 (0.41–0.45)

N denotes the total number of admissions in the RESPOND-COVID and TRIAGE cohorts and the number of test admissions in the 29K cohort, with the number in parentheses indicating the number of deaths. The results are based on the calibrated models and are presented as the means with 95% confidence intervals. AUC: area under the receiver operating curve based on test data. PPV: positive predictive value; NPV: negative predictive value; MCC: Matthews correlation coefficient.

## Data Availability

The datasets analyzed during the current study are not publicly available due to privacy considerations (data use agreements) and ethical restrictions. However, they can be made available from the corresponding author upon reasonable request and after obtaining the necessary approvals from the relevant authorities. The underlying code for this study, as well as the training and validation datasets, is not publicly available for proprietary reasons. However, qualified researchers may request access from the corresponding author, and they can be provided upon reasonable request.

## Supplementary Materials

The following supporting information can be downloaded at: https://www.mdpi.com/article/10.3390/jcm13216437/s1. Supplementary Figure S1: ROC curve for 10-day mortality; Figure S2: ROC curve for 30-day mortality; Supplementary Figure S3: ROC curve for 90-day mortality; Supplementary Figure S4: ROC curve for 365-day mortality.
