# Supplementary material for: Prospective and External Validation of Machine Learning Models for Short- and Long-Term Mortality in Acutely Admitted Patients Using Blood Tests
Source: J Clin Med. 2024 Oct 27;13(21):6437. doi: 10.3390/jcm13216437 (PMC11546962; doi:10.3390/jcm13216437)

Supplementary figure:

Sup. figure s1: ROC Curve for 10 days mortality

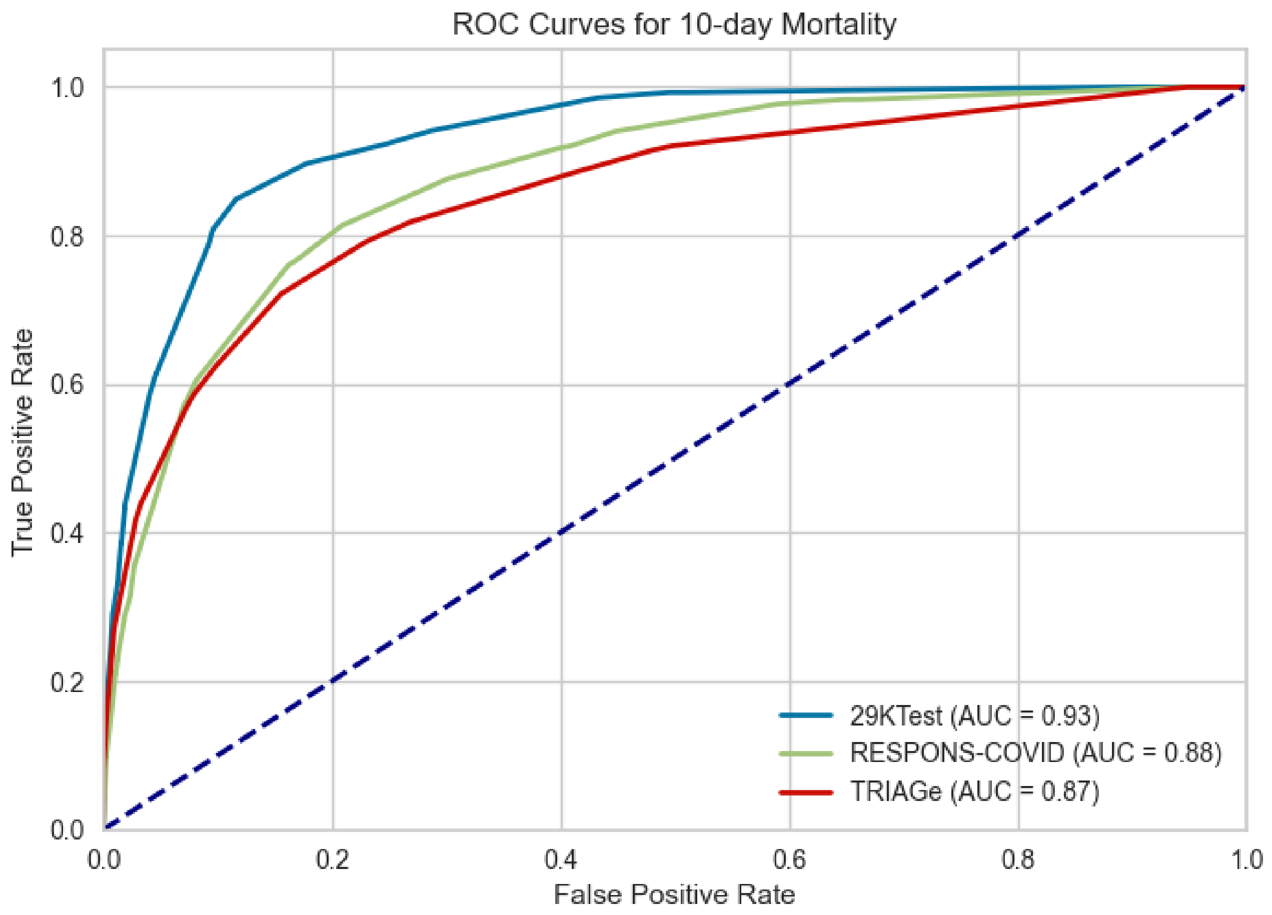

Sup. figure s2: ROC Curve for 30 days mortality

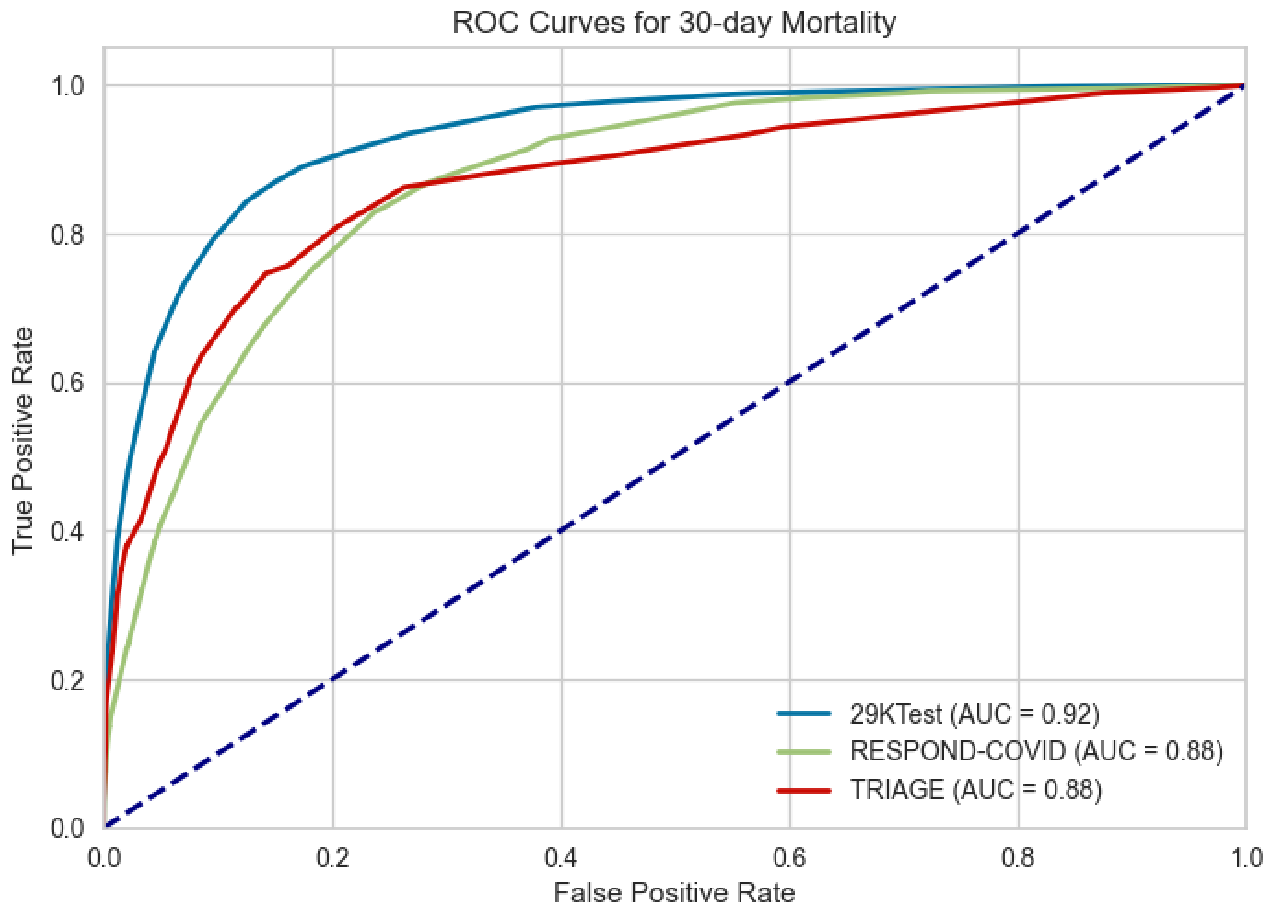

Sup.figure s3: ROC Curve for 90 days mortality

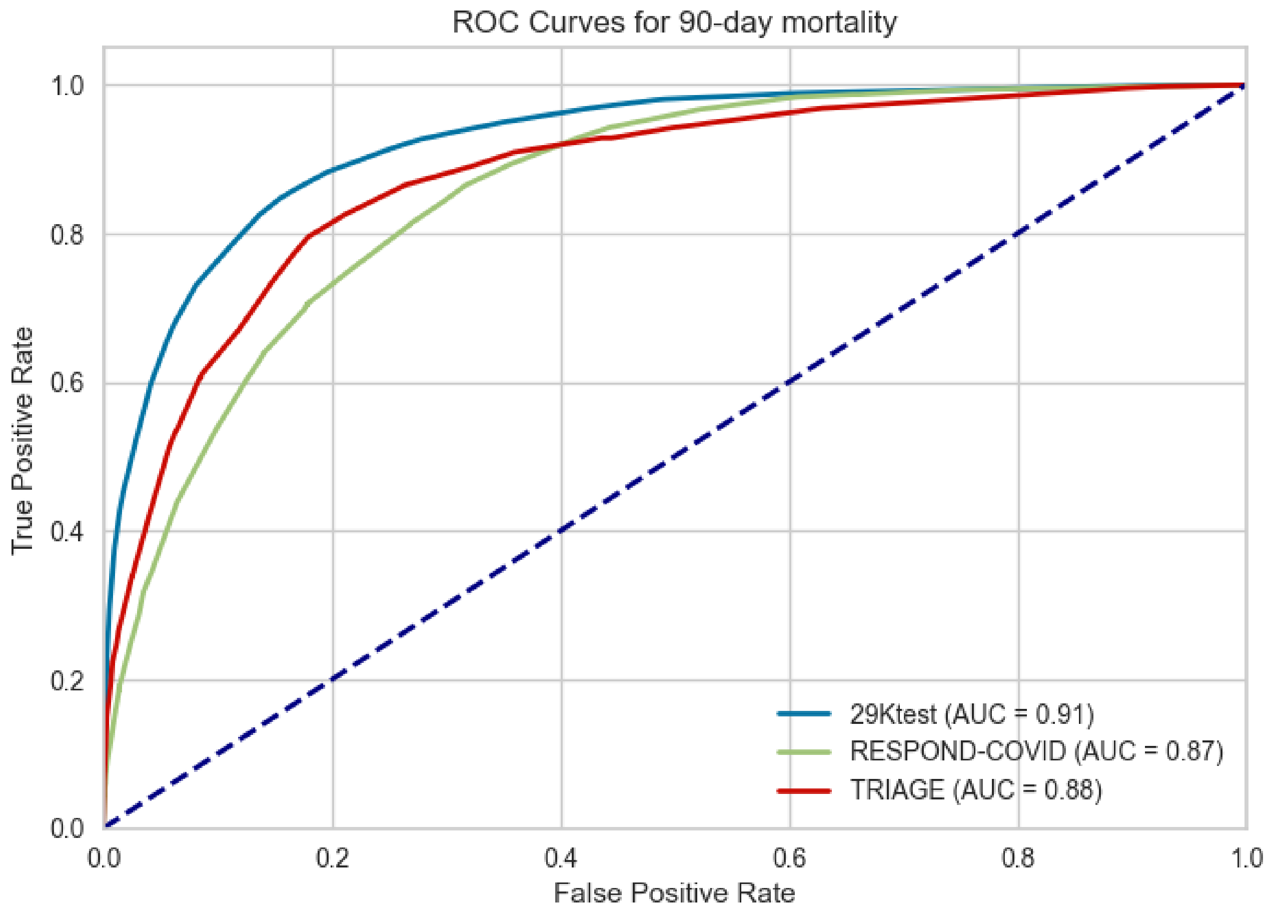

Sup. figure s4: ROC Curve for 365 days mortality

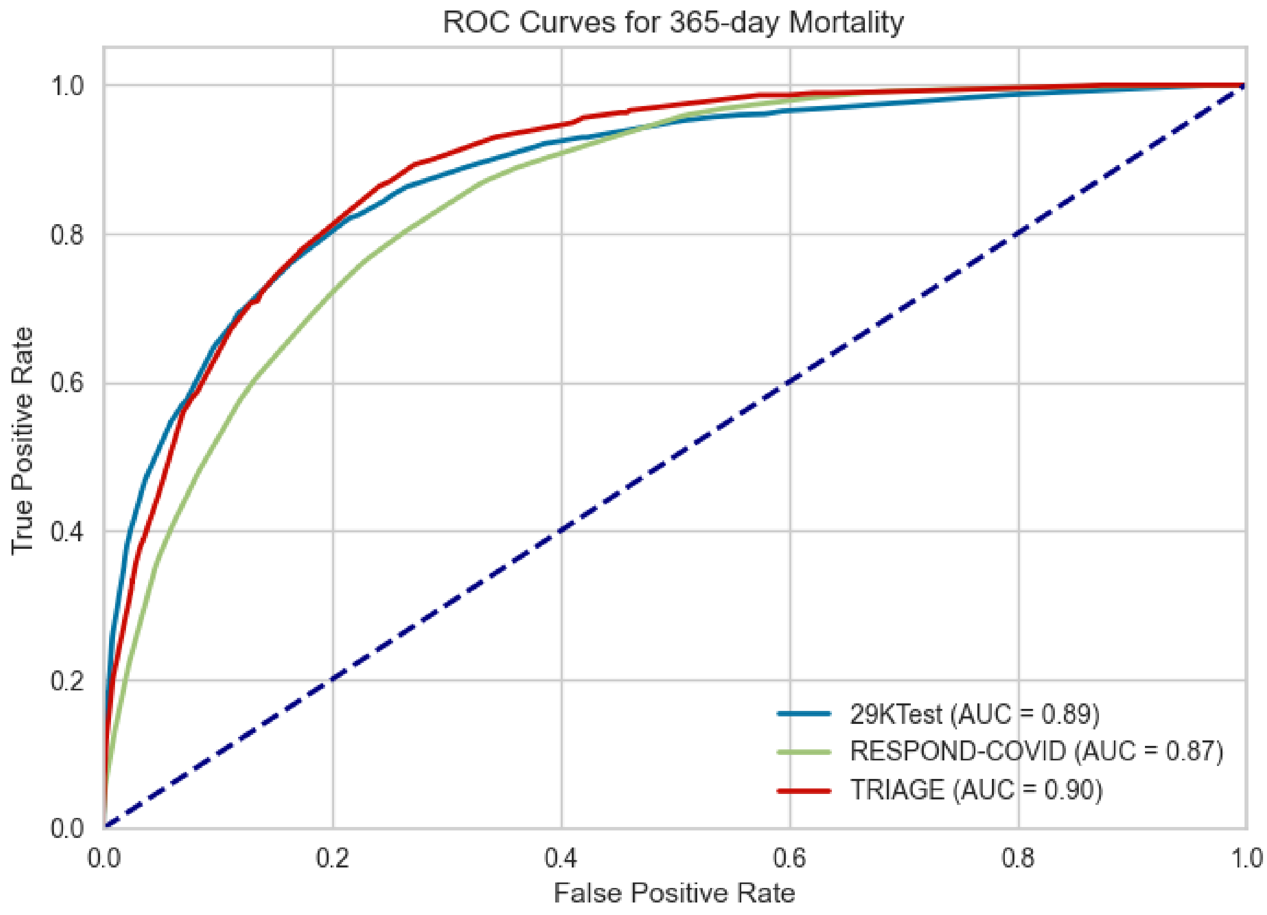

Supplement: Supplementary file 1 [file jcm-13-06437-s001.zip › jcm-3206870-supplementary.pdf]
